# Biomechanics-mediated endocytosis in atherosclerosis

**DOI:** 10.3389/fcvm.2024.1337679

**Published:** 2024-04-04

**Authors:** Jinxuan Wang, Jianxiong Xu, Tianhu Liu, Chaoping Yu, Fengcheng Xu, Guixue Wang, Shun Li, Xiaozhen Dai

**Affiliations:** ^1^ School of Basic Medical Sciences, Chengdu Medical College, Chengdu, China; ^2^ Department of Cardiology, The Third Affiliated Hospital of Chengdu Medical College, Chengdu, China; ^3^ School of Health Management, Xihua University, Chengdu, China; ^4^ Cardiology and Vascular Health Research Center, Chengdu Medical College, Chengdu, China; ^5^ Key Laboratory for Biorheological Science and Technology of Ministry of Education, State and Local Joint Engineering Laboratory for Vascular Implants, Bioengineering College of Chongqing University, Chongqing, China; ^6^ School of Biosciences and Technology, Chengdu Medical College, Chengdu, China

**Keywords:** atherosclerosis, endocytosis, biomechanics, lipid transportation, vascular cells

## Abstract

Biomechanical forces, including vascular shear stress, cyclic stretching, and extracellular matrix stiffness, which influence mechanosensitive channels in the plasma membrane, determine cell function in atherosclerosis. Being highly associated with the formation of atherosclerotic plaques, endocytosis is the key point in molecule and macromolecule trafficking, which plays an important role in lipid transportation. The process of endocytosis relies on the mobility and tension of the plasma membrane, which is sensitive to biomechanical forces. Several studies have advanced the signal transduction between endocytosis and biomechanics to elaborate the developmental role of atherosclerosis. Meanwhile, increased plaque growth also results in changes in the structure, composition and morphology of the coronary artery that contribute to the alteration of arterial biomechanics. These cross-links of biomechanics and endocytosis in atherosclerotic plaques play an important role in cell function, such as cell phenotype switching, foam cell formation, and lipoprotein transportation. We propose that biomechanical force activates the endocytosis of vascular cells and plays an important role in the development of atherosclerosis.

## 1 Introduction

Atherosclerosis, as the underlying mechanism of cardiovascular and cerebrovascular diseases, is caused by many complicated risk factors, such as lipid accumulation and abnormal biomechanics (1, 2). These disordered microenvironments in the vasculature result in vascular inflammation, excessive proliferation and migration, fibrosis, and extensive necrosis, which contribute to atherogenesis and the formation of vulnerable plaques (3). Biomechanics, as an emerging field of cell and developmental biology, is considered as a regulator of atherosclerosis through mechanosensitive channels. A recent study used the computed tomography angiography of human circumflex coronary artery to construct computational simulation model and revealed a causal link between low-density lipoprotein (LDL) transportation and wall shear stress in the coronary artery, in which flow patterns altered the influx and efflux of cholesterol (4). Moreover, endocytosis regulates the transportation and degradation of lipids and apoptotic cell debris in atherosclerotic plaques, which controls the interaction between cells and their microenvironment (5). Due to the important role of the mechanical environment and membrane trafficking in vascular dysfunction and atherosclerosis, it is important to investigate the changes in endocytosis induced by abnormal biomechanics.

Endocytosis-mediated intracellular transport and the positive regulation of signalling cascades are the key regulators of cholesterol homeostasis, which play a casual role in atherogenesis (6). Endocytosis and the subsequent intracellular itinerary are based on the encapsulation of the fluid plasma membrane, selective receptors and vesicle-associated proteins. This selective route allows endothelial cells (ECs) to act as a vascular barrier to regulate the transport of hydrophilic and hydrophobic substances in blood and prevent harmful substances in tissues (7, 8). Meanwhile, the influx and efflux of cholesterol are specifically controlled by endocytosis with lipoprotein receptors including low-density lipoprotein receptor family and scavenger receptor family (Table 1) (6, 9). Recent studies have revealed that the scavenger receptors-mediated transcytosis, which rely on caveolae-based intracellular vesicles, play an important role in the delivery and accumulation of LDL in artery (10, 11). The accumulation of LDL in the sub-endothelial area will further stimulate excessive uptake and exhausted metabolism of lipid in vascular smooth muscle cells (VSMCs) and macrophages, which will result in unexpected inflammation, polarization/phenotype transformation, autophagy and the formation of foam cells (12, 13).

**Table 1:** Lipoprotein-binding receptors during endocytosis in vascular cells.

Receptor	Cell type	Ligands relevant to lipoprotein	References
LDLR	Macrophage, EC, VSMC	LDL (APOB and APOE)	(14)
LRP1	Macrophage, EC, VSMC	LDL	(15)
LRP5/6	Macrophage, EC, VSMC	LDL	(16)
LRP8	Macrophage, EC	VLDL	(17)
VLDLR	Macrophage, VSMC	VLDL	(18)
CD36	Macrophage, EC, VSMC	LDL, HDL, acLDL, oxLDL, VLDL	(19, 20)
LOX1	Macrophage, EC, VSMC	oxLDL	(21)
SR-B1	Macrophage, EC	HDL, LDL, VLDL, acLDL, oxLDL	(11, 22)

During the development of atherosclerosis, biomechanics, including shear stress, tensile force, and stiffness, play an important role in vascular inflammation, oxidative stress and lipid transportation (23, 24). Low shear stress (LSS) and oscillatory shear stress (OSS) generated by disturbed flow have been identified as hazard factors of atherosclerosis in hypercholesterolemic mini-pigs that correlate plaque growth with vulnerable features (25). These LSS and OSS will result in unexpected uptake of LDL and promote the development of atherosclerosis (26). Meanwhile, studies have also revealed that tensile force activation of stretch-related ion channels clinically contributes to arterial remodeling and relevant vascular dysfunction (27, 28). Moreover, recent research has also suggested that arterial stiffness is associated with artery atherosclerosis stroke in human with GWAS analysis that comprises 127,121 individuals of European ancestry (29). Thus, it is important to discuss the relationship between biomechanics and endocytosis in atherosclerosis.

In this review, we summarized the major types of endocytosis in atherosclerosis, including clathrin-mediated endocytosis, caveolae-mediated endocytosis, phagocytosis and micropinocytosis. We then focused on the role of endocytosis in responding to biomechanical forces and describe the signal transduction involved in lipid transportation in the biomechanical microenvironment. The integration of biomechanics and endocytosis might improve our understanding of the transportation of high-risk lipoprotein and contribute to a better understanding of the formation of atherosclerotic plaques.

## 2 General mechanisms of endocytosis in atherosclerosis

**Figure 1:**
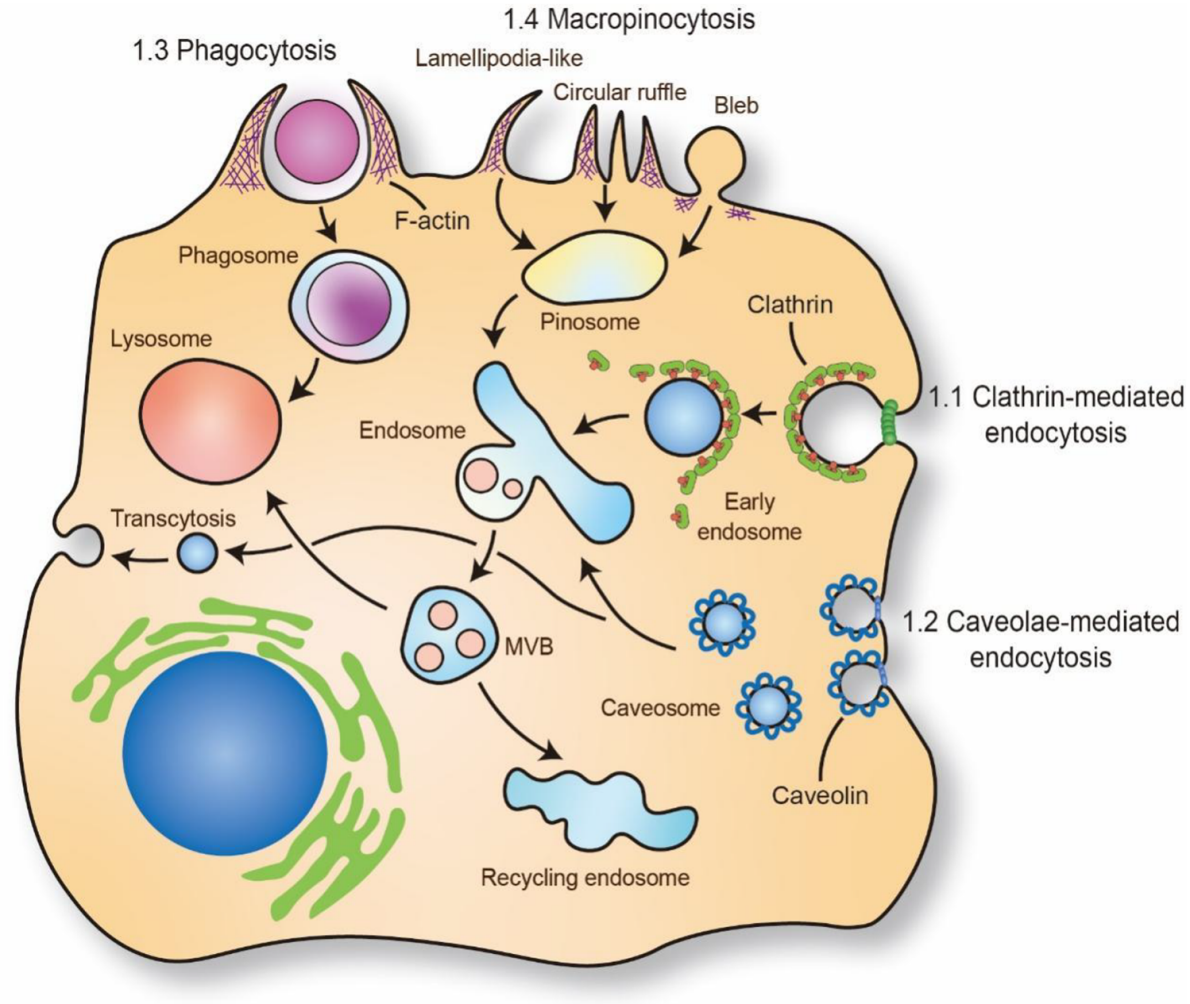
Mechanism of molecules and macromolecules trafficking through endocytosis. The putative endocytic portals in atherosclerosis mainly includes phagocytosis, micropinocytosis, clathrin-mediated endocytosis, caveolae-mediated endocytosis. The phagocytosis and micropinocytosis are driven by the reorganization of cortical cytoskeleton with the formation of elaborate membrane protrusions. Clathrin-mediated endocytosis and caveolae-mediated endocytosis require the recruitment of clathrin and caveolin on plasma membrane to form “coated pit” and vesicle and internalize cargo. The trafficking cargo will transport to endosome and lysosome. Caveolae-mediated endocytosis also regulate transcytosis in ECs which is important for maintaining the barrier of vascular. MVB: multivesicular bodies. The schematic diagram was made by the authors using Adobe Illustrator 2021.

Endocytosis, as an important part of the cellular trafficking system, is a conserved channel for internalizing molecules and macromolecules through membrane deformation (30). The typical process of endocytosis contains four fundamental steps: (1) molecules specifically bind to receptors or the cell membrane; (2) coating proteins, cytoskeleton and membrane fusion proteins are rearranged to drive cell membrane-encapsulating molecules; (3) molecules are encapsulated in trafficking vesicles, such as endosomes and phagosomes; and (4) vesicles are transported to subcellular organelles (Figure 1). Due to the entry mechanism, endocytosis can be classified into pinocytosis and phagocytosis. The pinocytosis can further be classified into clathrin-mediated endocytosis and clathrin-independent endocytosis, such as caveolae-mediated endocytosis, caveolae- and clathrin- independent endocytosis and macropinocytosis, which can form differently sized vesicles (31) (Figure 1). Meanwhile, some specific inhibitors are also widely used to detect the influence of endocytosis to cell functions (Table 2). These engulfment processes drive lipid transportation and apoptotic cell clearance in the vasculature, which are inexorable events in atherosclerosis (37, 38). Here, we briefly discussed the role of different endocytosis mechanisms in atherosclerosis.

**Table 2:** Types and effects of endocytic inhibitors.

Inhibitor name	Endocytosis type	References
Dynasore	Dynamin-dependent endocytosis	(32)
methyl-β-cyclodextrin	Caveoae-mediated endocytosis	(33)
filipin	Caveoae-mediated endocytosis	(34)
chlorpromazine	Clathrin-mediated endocytosis	
Pitstop 2	Clathrin-mediated endocytosis	(35)
Wortmannin	macropinocytosis	(36)
LY294002	macropinocytosis	
Amiloride	macropinocytosis	

### 2.1 Clathrin-mediated endocytosis

Clathrin-mediated endocytosis that molecules are packaged by a clathrin coat by clathrin triskelia based polymerization, can internalize and transport proteins, lipids, hormones and metabolites (39). The formation of clathrin-based vesicles requires the recruitment of adaptor proteins (APs) on the plasma membrane to initiate and construct clathrin-coated pits (40). Meanwhile, this binding process of APs is mediated by transporting receptors, plasma membrane-specific lipid phosphatidylinositol-4,5-bisphosphate, EGFR pathway substrate 15, and epsin proteins, which supports the assembly of clathrin-coated vesicles (41–44). Moreover, clathrin-coated pits are scissored via the enzyme dynamin recruited by BAR domain-containing proteins and form individual clathrin-coated vesicles (45, 46). Finally, the clathrin coat is disassembled and recycled from vesicles to allow the vesicle to fuse with endosomes (Figure 1) (47).

AP1 binds membranes enriched for phosphatidylinositol 4-phosphate, such as the trans Golgi network, while AP2 associates with phosphatidylinositol 4,5-bisphosphate of the plasma membrane. At their respective membranes, AP1 and AP2 bind the cytoplasmic tails of transmembrane protein cargo and clathrin triskelions, thereby coupling cargo recruitment to coat polymerization (48). Under physiological conditions, EGFR is internalized mostly by clathrin-mediated endocytosis. Growth factor binding to EGFR accelerates its internalization through clathrin-coated pits which is followed by the efficient lysosomal targeting of internalized receptors and results in receptor down-regulation. A recent study revealed that clathrin-mediated endocytosis was involved in the uptake of oxidized low-density lipoprotein (oxLDL) in macrophages through protease-activated receptor trafficking (49). Moreover, knockout of espins, which are important in clathrin-mediated endocytosis in myeloid cells, reduced the uptake of oxLDL through LRP1 and reduced the process of atherosclerosis (50). These results suggested that clathrin-mediated endocytosis, as an important cargo transporter, was involved in the progression of atherosclerosis.

### 2.2 Caveolae-mediated endocytosis

Caveolae, as the most abundant features in lipid rafts that invaginate to initialize bulb-shaped caveolar pit and form 60–80 nm specialized vesicles, are considered to participate in many biological functions, such as lipid regulation, material transportation, and signal transduction (51). The start and maintenance of caveolae-mediated endocytosis rely on caveolins, which are coated on the membrane surface of vesicles for structural formation (52). Generically, caveolin-1 (Cav1) and caveolin-2 are expressed in non-muscle cells, and caveolin-3 is expressed in some smooth muscle cells, which are anchored to the cell membrane and compose caveolae (53). Moreover, the caveolar structure contains approximately 144 molecules of caveolin that can bud off to construct endocytic caveolar vesicles and fuse with the caveosome and endosome, which can transport various cargo, such as lipid droplets and fatty acids (52, 54, 55). Recent studies have found that caveolae-based endocytosis is contributed to internalize LDL and VLDL in EC and macrophages. Silence the structural protein Cav1 of caveolae in Ldlr^−/−^ mice will dramatically decrease the accumulation of lipids in the vasculature and reduce atherosclerotic plaque (56, 57). Cav1 is also responded shear stress and increases flow velocity in artery and reduces vascular inflammation and macrophage infiltration in disturbed flow area (56). Meanwhile, the loss of Cav1 in ECs reduces the endocytosis and transcytosis of LDL and disturbs autophagy. However, the relationship between autophagy and caveolae-mediated endocytosis is mutually regulated, and the loss of one of them will affect the functional process of the other (58, 59). Recent study has also revealed that LDL particles are colocalized with endothelial SR-B1 and cross endothelial cell barrier through caveolae-mediated endocytosis (11). These results suggested that caveolae-mediated endocytosis in vascular is closely related to the transport of LDL in ECs and accelerating atherosclerosis.

### 2.3 Phagocytosis

Phagocytosis, defined as the uptake of particles larger than 0.5 μm through plasma membrane encapsulation, is important in eliminating apoptotic cells, bacteria and other foreign materials in macrophages and other phagocytes (60). Typically, phagocytosis occurs in immune cells such as macrophages, monocytes, microglia and neutrophils and is activated by specific receptors in the cell membrane to recognize particles. Particles are then encapsulated by a cup-shaped membrane and transported in phagosomes, resulting in progressive degradation of cargo. Meanwhile, non-professional phagocytes (such as epithelial cells and ECs) also participate in the clearance of apoptotic cells and particles through phagocytosis and are associated with the recruiting role of macrophages (61, 62). The progression of atherosclerosis is associated with the excessive accumulation of apoptotic cells, including macrophages and VSMCs, and sufficient phagocytosis, resulting in the formation of a necrotic core and rupture plaque (63). Macrophages deactivate efferocytosis-related signalling such as ERK5, Rac2 that involve in cytoskeleton remodeling will lead to losing phagocytic capacity and accelerate atherosclerotic plaque formation (64, 65). Meanwhile, lack of phagocytosis will further promote the transition of VSMCs to macrophage-like cells through activating KLF4 and exacerbating the instability of atherosclerotic plaque (66). Yoko et al. found that blocking the “don’t eat me” molecule CD47, which is activated by TNF-α via NFKB1 and important for avoiding phagocytosis from phagocyte, can help macrophages to recognize foam or apoptotic cell and stimulate efferocytosis to promote phagocytic clearance and reduce atherosclerotic plaque (38). Furthermore, recent study has revealed that the enhancing phagocytosis of macrophages by CD47 relies on LRP1 to internalize during atherosclerosis. Loss LRP1 in macrophages will decrease the blocking effect of CD47 on efferocytosis and promote the formation of necrotic core in atherosclerotic plaque (67).

### 2.4 Macropinocytosis

Macropinocytosis is a non-selectively endocytic process for engulfing fluids and particles by forming vacuole-like extensions of the plasma membrane, which is an important route for the degradation of lipids, proteins in the extracellular matrix and dead cells (68). Macropincytosis, as an actin-dependent endocytic process, starts with ruffing across and encapsulating the plasma membrane, including protrusion, folding, and closure on the cell surface (69). In atherosclerosis, recent studies have revealed that macropinocytosis is responsible for engulfing LDL in macrophages and VSMCs and contributes to the formation of foam cells (36, 70). Meanwhile the engulfing enzyme-modified LDL but not acetylated or oxidized LDL through calcium dependent macropinocytosis is a potent role in forming VSMC derived foam cell (36). These lipid-overlapping foam cells in the vascular wall play an important role in the development of the necrotic core and late-stage atherosclerosis.

## 3 Shear stress on endocytosis

**Figure 2:**
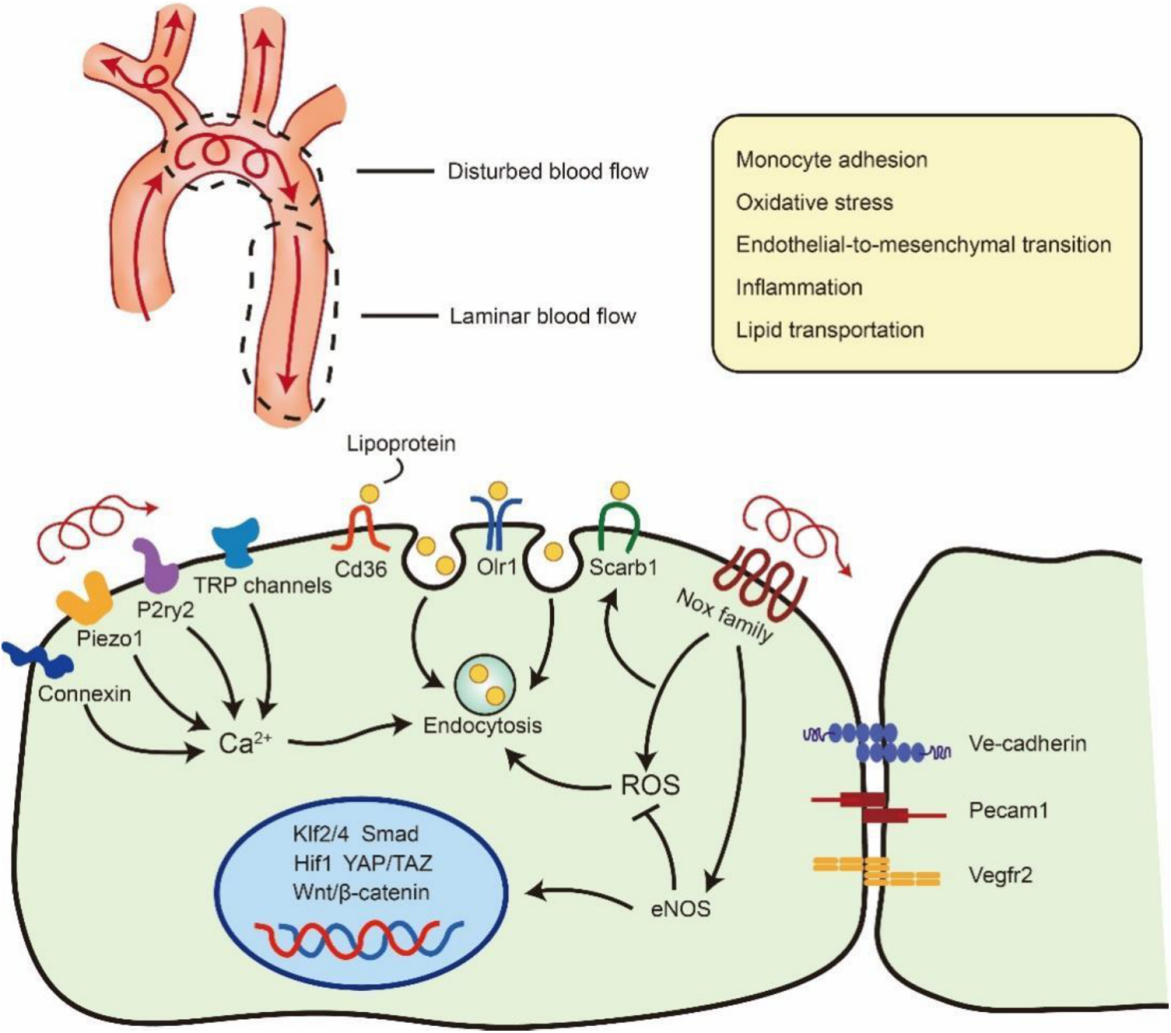
Flow regulation of lipoprotein uptake via endocytosis in ECs. Blood flow at branches and bends of arteries will be disturbed and generate LSS or OSS. The unexpected OSS in artery will result vascular inflammation, oxidative stress and so on. OSS also can stimulate Ca^2+^ channel and Nox family to promote Lipoprotein endocytosis in atheroprone area. Meanwhile the disturbed flow will down-regulate the expression of eNOS and further influence the transseptal manner of ECs such as Klf2/4, Smad, Hif1, YAP/TAZ, Wntβ/-catenin. ROS: reactive oxygen species. eNOS: endothelial nitric oxide synthase. The schematic diagram was made by the authors using Adobe Illustrator 2021.

Shear stress is a blood flow-generating frictional force that is closely associated with vascular dysfunction and atherosclerosis. Several studies have demonstrated that atherosclerotic plaque usually emerge in near branches and bends of arteries that are exposed to disturbed flow, generating LSS or OSS (71, 72). These blood flow-induced vascular dysfunctions are linked to the changes of several signalling pathways, such as Klf2/4, Hippo–Yap–Taz, and Wnt/β-catenin, which participate in maintaining vascular integrity and tissue homeostasis (72–74). A recent study has revealed that shear stress not only mediates signal transduction but also increases LDL coverage on the endothelial glycocalyx, which controls the transportation of LDL across the vascular wall (75). Thus, it is essential to discuss the influence of shear stress on cell endocytosis during atherosclerosis (Figure 2).

ECs, as the barrier of vessel and blood flow, are equipped with various mechanosensitive channels that transfer biomechanical signals to modulate cellular function and behaviour, such as inflammation, endothelial-to-mesenchymal transition, and endocytosis (2). These shear stress-sensitive signals are mostly membrane receptors, cell‒cell junctional proteins and cell-matrix adhesion proteins (76). Moreover, Exposure of ECs on 10 dynes/cm^2^ shear stress will result an increased endocytosis and can be reduced by inhibiting reactive oxygen species (ROS) (77). Meanwhile, the accumulation of NO and ROS in LSS or OSS areas will result in the increased stabilization and expression of Cav1, which may enhance Cav1-mediated transcytosis (78). The surface density of caveolae in the cell membrane, which has been implicated in haemodynamic forces, is necessary for mechanotransduction and arterial remodelling (79). Ramírez et al. found that Cav1 is highly expressed in the “athero-prone” area and controls the transcytosis of LDL in atherosclerosis. Moreover, mice lacking Cav1 expression show less accumulation of lipids in athero-prone areas and atherosclerotic plaques (56). Our laboratory's recent study has revealed that LSS- and OSS-induced ROS can promote the internalization of extracellular vesicles in vascular ECs. We also observed that extracellular vesicles accumulated in the artery arch that underwent LSS and OSS (80). Abnormal shear stress can also activate multiple Ca^2+^ channels, including Piezo1, P2ry2, connexin and transient receptor potential (TRP) channels, which are considered mechanosensors and are significantly associated with inflammation in ECs during atherosclerosis (72, 81, 82). Meanwhile, Ca^2+^ influx can initiate activity-dependent bulk endocytosis and participate in the engulfment of LDL (83, 84).

Ongoing studies of OSS and LSS in ECs also report the role of mechano-transduction in endocytic receptors. Due to numerous researchers have performed transcriptome data under different shear stress, these analyses reveal that shear stress can mediate multiple transcriptional processes of endocytic receptors, such as Cd36 and Scarb1 (85). The overexpression of these lipoprotein receptors in arteries promotes the uptake of macromolecules into cells, which participates in the formation of atherosclerosis (6). Meanwhile, shear stress also plays an important role in the reorganization of Cdc42-dependent actin polymerization, which is important for LDL endocytosis (86, 87). Macrophage cortical F-actin depolymerization is required for actin polymerization to form a hydrolytic compartment-the lysosomal synapse, which digests aggregated LDL via Cdc42 Rho GTPase and GEF pathway. In summary, shear stress regulates multiple processes of endocytosis that control the accumulation of LDL and other macromolecules in the vasculature, thereby critically contributing to the development of atherosclerosis near branches and bends of arteries.

## 4 Stretch and tension force on endocytosis

**Figure 3:**
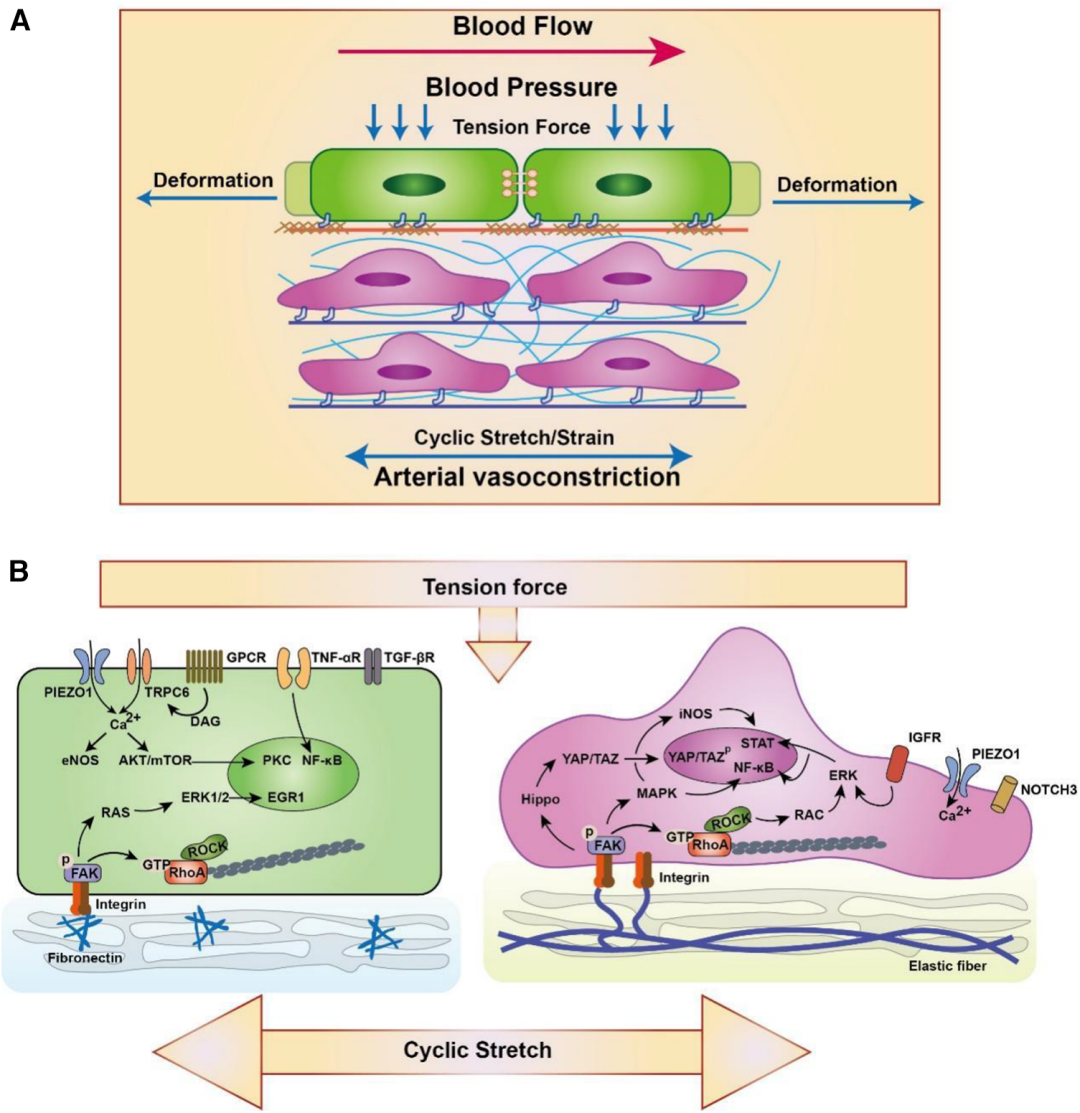
Schematic diagram of EC (left) and SMC (right) responding to the high strain/stretch and hypertension. (**A**) ECs are from the intima and VSMCs are form the media and adventitia. Blood pressure is perpendicular to the artery, which results in circumferential stretching and deformation of artery. Meanwhile, the physiological extension or arterial vasoconstriction derived from VSMCs also play an important role in the circumferential stretching of artery. (**B**) Biomechanics are sensed by varieties of membrane mechanoreceptors in artery such as PIEZO1 (28, 90), TRPC6 (90), GPCR (90), TNF-αR (91), TGF-βR (92), IGFR (93) and NOTCH3 (94). Meanwhile, ECs and VSCMC can connect with the extracellular matrix through focal adhesion complex to response and sustain stress (95). Focal adhesion complex contain integrin and FAK bind to ECM and is associated with multiple signaling transduction including ERK/MAPK (96–98), Hippo/YAP (99), RhoA (100) and so on (88). Furthermore, Ca^2+^ and other ionic signaling also participate in the mechano-transduction and involves in the transcriptional regulation (101). The schematic diagram was made by the authors using Adobe Illustrator 2021.

ECs and VSMCs are sensitive to changes in physiological mechanical stretch and tension force that contain circumferential and axial stresses of approximately 100 kPa (1 × 10^6^ dyne/cm^2^) from periodical distention and relaxation in arteries (88). Arteries under hypertension also inherit axial stress, which is associated with blood pressure and periodic distention and relaxation of arteries (89) (Figure 3). Meanwhile, VSMCs can be the engine of artery showing stronger contractile ability to generate and sustain stress on artery that is important in maintaining vessel tone and blood pressure (102). Elastic lamellae comprising elastin and collagens, which attaches and sandwiches VSMCs in the middle through focal adhesion complex, affects VSMCs contraction and relaxation (103, 104). Furthermore, ECs and VSMCs subjected to cyclic stretch display an elongated spindle morphology and show reorganization of the cytoskeleton. Studies have defined that 5%–10% strain is the physiological stretch, while excessive strain (15%–20%) is the pathological stretch in artery (105, 106). The unexpected stretch and tension force on vascular cells including ECs and VSMCs will activate multiple signalling and result excessive proliferation, migration and apoptosis (Figure 3) (107, 108). The VSMCs response to cycle stretch can express integrin αVβ3, which can inhibit ox-LDL-induced apoptosis although PINCH-1 in the progression of atherosclerosis (109, 110). Moreover, the cardiovascular cells response to cyclic stretch increase the accumulation and update of extracellular matrix (111). These studies suggest that normal physiological stretch and tension are important signals for cardiovascular function and contribute to the homeostasis and development of vessels.

**Figure 4:**
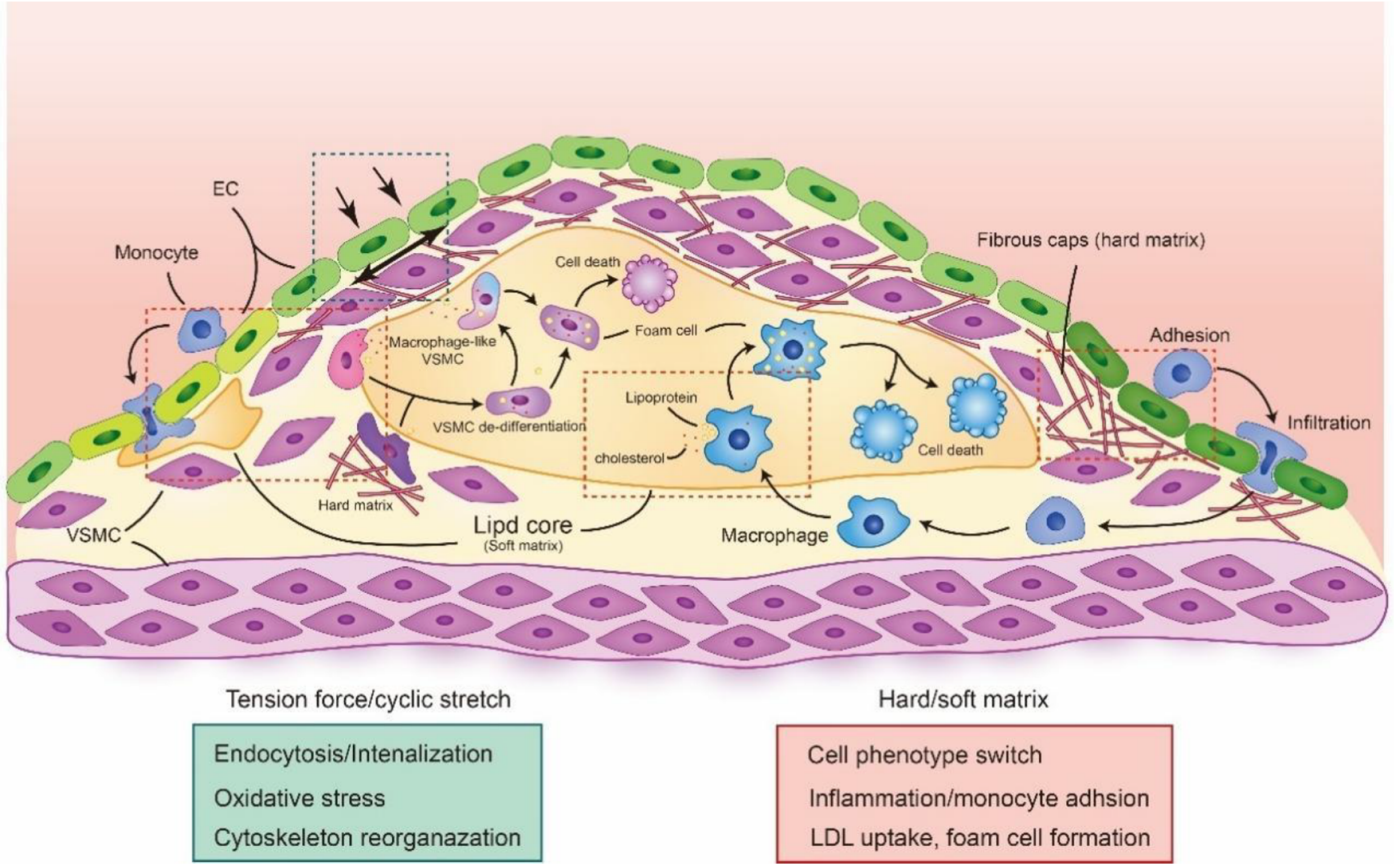
The biomechanical forces in atherosclerotic plaque. The alteration of biomechanical forces in atherosclerotic is important in plaque development and progression. The cyclic stretch and tension force will be induced by the changes of atherosclerotic plaque, which regulate multiple cell function. Meanwhile the changes in plaque composition and architecture also result the alteration of cell stiffness, making cell phenotype switching, inflammation and foam cell formation. The red rectangle is highlighted the biological process under hard or soft matrix and green rectangle is highlighted the biological process under tension force or cyclic stretch. The schematic diagram was made by the authors using Adobe Illustrator 2021.

Pressure-derived stretch and tension force, which derived from the vascular deformation caused by blood pressure on the vessel wall, can influence the transportation of albumin and LDL. Studies have used 4 mm, 5 mm and 6 mm sleeves to restrict vessel from in New Zealand White rabbit and pressurized at 70, 120, or 160 mm Hg blood pressure, which control the deformation of vessel. The results showed that the accumulation of LDL in vessel was increased between 120 and 160 mm Hg with 5 mm sleeves, which explains the relationship between pressure-derived stretch and atherosclerosis (112). Stretch force also promotes the oxidation of LDL and accelerates the accumulation of ox-LDL in VSMCs (113). Meanwhile, cells stimulated with cyclical tensile stretch highly express the ox-LDL receptor Lox1, which is essential for internalizing ox-LDL (114). Moreover, membrane tension is also an important regulator of clathrin-mediated endocytosis through controlling the formation of clathrin-coated pits. Joseph et al. found that high tension interrupts the process of flat membrane-to-clathrin-coated structure transition by inhibiting the recruitment of epsin to the plasma membrane (44). Moreover, a recent study found that Torc2, a rapamycin-mediated protein kinase, can regulate plasma membrane tension to affect the reorganization of the actin cytoskeleton and vesicle fission to endocytosis sites (115). These results suggest that stretch and tension forces are essential for endocytic processes that regulate the transportation of multiple lipids and proteins in the vasculature (Figure 4).

## 5 Stiffness on endocytosis

Stiffness, also known as elasticity, is the mechanical force to resist deformation and is important for cell proliferation, migration and signal transduction. Arterial stiffness is closely associated with degenerative and remodelling changes in the extracellular matrix, resulting in vascular calcification, hypertension, and atherosclerosis (116). A recent Multi-Ethnic study with 6,814 men and women aged 45–84 years found that high load-dependent carotid artery stiffness is associated with a higher incidence of subclinical atherosclerosis and thus contributes to multiple cardiovascular events, such as stroke and hypertension (117). The stiffness of the artery is determined by their physiological conditions during atherosclerosis, such as the lipid core/necrotic core (1 kPa), fibrous plaque (35.5–54 kPa) and calcification (80–300 kPa), compared with normal artery (10–50 kPa) (118, 119). Meanwhile, the changing matrix stiffness also mediates the mechanical signal transduction and phenotypic transformation of vascular cells, which is closely related to cell fate and vascular function (118, 120). Recent studies have revealed that changing matrix stiffness in the vasculature promotes the adhesion of monocytes on ECs by enhancing the expression of miR-126 (targeting VCAM-1) and miR-222 (targeting ICAM-1) (121). Meanwhile, 1 kPa (soft) and 100 kPa (hard) substrate stiffnesses also increase cholesterol efflux in VSMCs and increase the expression of the macrophage marker CD68, suggesting that substrate stiffness can regulate the phenotypic switching of VSMCs (Figure 3) (122).

Endocytosis, as an important route of lipid metabolism, can also be regulated by cell and matrix stiffness. The surface topography of substrates regulates cell stiffness by activating mechanical signalling pathways. Li et al. found that a nanostructure stiff substrate can alter cell stiffness and behaviour to enhance clathrin-dependent endocytosis through its nano-topographical effect on integrin receptors. It has also been revealed that cells on a glass-based nanostructure stiff substrate respond similarly to 1 kPa soft hydrogels, which can reduce cell stiffness and membrane tension force (123). Furthermore, the cytoplasmic stiffness that influences deformability and membrane invagination can modulate the endocytic ability of cells. The high cortex stiffness of the subcellular structure will affect cell deformability, resulting in less phagocytic ability on macrophages (124). Because the stiffness of the necrotic core and lipid core in atherosclerotic plaques are nearly 1 kPa, we suspect that cells in atherosclerotic plaques will engulf more lipoprotein and apoptotic cells, resulting in the formation of foam cells.

A recent study revealed that macrophages prefer to take up native LDL and ox-LDL on 1 kPa soft substrates but with no difference in proliferative activity on soft and stiff substrates in an inflammatory microenvironment (125). However, Li et al. found that macrophages on soft 4 kPa PA hydrogels promote cell apoptosis and have less ox-LDL phagocytosis than those on 30 kPa substrates (126). Moreover, TRPV4 calcium-permeable channels that respond to matrix stiffness promote the transmembrane transport of Ca^2+^ and regulate the endocytosis of ox-LDL although CD36 (127). They also found that TRPV4, known as a mechano-sensor, plays an important role in regulating macrophage foam cell formation (127). Furthermore, Cav1 as the important part of caveolae will stimulate by soft substrates to modulate YAP activity through controlling actin polymerization and mediate caveolae internalization (128). These mechanotransducting property of Cav1 is also associated with cell stiffness and caveolae-based endocytosis. Le et al. discovered that disturber flow will increase the elastic modulus of ECs by increasing the expression of Cav1, which is positive correlation with the uptake of oxLDL (129). Recent studies have also revealed that the membrane tension force is important for Cav1-based vesicular trafficking, which is regulated by the stiffness of the extracellular matrix and depends on the mechano-transduction of the integrin/RhoA axis to stimulate a Cav1-dependent manner (130). These studies suggest that matrix and cell stiffness may regulate the transportation of lipids and proteins, which may contribute to the progression of atherosclerosis (Table 3).

**Table 3:** The influence of biomechanics on endocytosis.

Biomechanics	Endocytosis	Biological function	References
Shear stress	Caveolae-mediated endocytosis	Regulate the expression of Cav1 and the distribution of caveolae	(78, 79)
	Clathrin-independent endocytosis	Reorganize Cdc42 dependent actin polymerization and promote LDL endocytosis	(86)
	Endocytic receptors	Regulate the expression of endocytic receptors (Cd36, SR-B1)	(85)
Stretch/tension force	clathrin mediated endocytosis	Membrane tension regulate the formation of clathrin coated pits	(44)
	Endocytic receptors	Cyclic tensile stretch regulates the expression of endocytic receptor-LOX1	(114)
Stiffness	Clathrin dependent endocytosis	nanostructure stiff substrate can alter cell stiffness to regulate endocytosis	(123)
	Endocytosis	Cytoplasmic stiffness regulate cell deformability to modulate endocytosis	(124)
	Endocytosis	Soft matrix promotes the uptake of ox-LDL in macrophages	(125)
	Endocytosis	Matrix stiffness regulate the endocytosis of ox LDL through stimulating TRPV4 calcium permeable channels	(127)
	Caveolae-mediated endocytosis	Substrate stiffness regulates the distribution of caveolae and modulates vesicular trafficking	(130)

## 6 Endocytosis for nanomedicine in atherosclerosis

Nanomedicine have widely applied in detecting and treating atherosclerosis with designing multiple responded or targeting molecule. Whereas, there are few researches focusing on the transporting route of nanoparticles into cells during treatment. Researchers have considered that nanoparticles firstly will closely contact with targeted cells and induce cell membrane to generate forces. Then, cell membrane will further encapsule nanoparticles through endocytic route and internalize nanoparticles (131). Thus, according to the endocytic route, we can design engineered nanoparticles to target diseased cells or even loading nano-drugs into living cells to assist drug delivery (132). Hu et al. design a tetrapod needle-like PdH nanozyme that can be internalized and storage into macrophages. By using the inflammatory response property, macrophages as the vesicles can deliver the nanozyme to atherosclerotic area and inhibit ROS (133). Furthermore, the biomechanics also can influence the endocytic process. Qin et al. have found that disturbed flow in artery will accelerate the internalized process of nano-sized extracellular vesicles through activating ROS in ECs (80). Due to the important role in avoiding the phagocytosis from immunity, researchers have used macrophages-membrane to encapsule nanoparticles and can efficiently and safely inhibit the progression of atherosclerosis (134). Thus it is important to develop nanomedicine based on the mechanism of intracellular endocytosis.

## 7 Discussion

The mechano-environments of atherosclerotic plaques are complex and involve multiple factors for vascular homeostasis that control cell transcription and biological function. Endocytosis is a fundamental process in which vascular cells internalize nutrients, lipids and other molecules. However, the unexpected endocytosis of lipids and inflammatory molecules accelerates atherosclerosis via lipid accumulation and the formation of foam cells (56, 135). It is urgent to fully elucidate the interactions between biomechanical force and vascular endocytosis. Recent studies of the single-cell transcriptome and functional screening of membrane receptors have already revealed the role of biomechanics in vascular cell endocytosis in atherosclerosis. They presented a comprehensive single-cell atlas of all cells in the carotid artery under d-flow, identified previously unrecognized cell subpopulations and gene expression signatures. Long-term exposure of ECs to low laminar shear stress leads to enhanced Endoglin expression and endocytosis of Endoglin in Cav1-positive early endosomes, highlighting Cav-1 vesicles as a SMAD signaling compartment in cells exposed to low atheroprone laminar shear stress (85, 136). However, a challenge remains regarding how the signal transduction of biomechanical force to endocytosis in atherosclerosis is regulated via different pathways, responsive molecules and endocytosis-related proteins. Nevertheless, some researchers have explored whether biomechanical force regulates multiple pathways that are associated with mechanosensitive transcription factors that typically participate in the transcription of lipoprotein-transported proteins (137, 138). Moreover, cells are prefer to engulfing stiff nanoparticles which can easily achieve full wrapping (131). The size and stiffness of LDL also will decrease in acidic condition or oxidation which suggests cells may hard to engulf LDL particles (139, 140). Therefore, it is important to identify novel mechanosensitive pathways and dynamic processes of endocytosis in arteries.

The material transported by endocytosis is also essential for nanomedicine, which provides a targeted route for nanoparticles to enter and deliver cargo in cells. Due to the intracellular delivery of nanoparticles relying on vascular physiology, microenvironment and cell phenotype, it is important to select and design suitable nanomaterials to deliver drugs. Several studies of nanomedicine have found that the accumulation of nanoparticles in cells is influenced by the biomechanical environment in the vasculature. The engulfment of nanoparticles is associated with the shear stress of the vessels, and physiological changes in ECs under disturbed flow increase the accumulation of nanoparticles (141). Meanwhile, disturbed flow inducing oxidative stress can also promote the uptake of nanoscale materials (80). This flow-dependent accumulation is important for drug delivery during atherosclerosis, which is usually present at the vascular branch and downstream of curved regions. Researchers have also found that physiological cyclic stretch promotes the internalization of silica nanoparticles, which is related to cell stress and exocytotic events (142). Moreover, the changes in plasma membrane morphology by cyclic stretch will affect the distribution of actin and reduce the internalization of nanoparticles in VSMCs (143). Thus, understanding the role of nanoscale materials in endocytosis is important for investigating intracellular delivery and providing more targeted points for nanomedicine.

Overall, biomechanical force is an essential feature to regulate endocytosis that accommodates molecule and macromolecule trafficking and lipid metabolism in atherosclerosis. Endocytosis is not only important for nutrient uptake but also a primary route by which lipid particles enter cells. Here, we summarized the main endocytic process in atherosclerosis and described the interaction role of biomechanics and endocytosis in atherosclerosis. A consistent conclusion from these studies is that the changing biomechanics of the vasculature will result in disordered endocytosis, which is typically associated with lipoprotein transportation. Therefore, further analysis of pathophysiological endocytosis under biomechanical force will improve our understanding of the development of atherosclerosis and lead to the discovery of new therapeutic drugs and targets in atherosclerosis.
